# Upon the Efficacy and Mode of Administration of Belladonna and Atropia

**Published:** 1847-01

**Authors:** W. R. Wilde

**Affiliations:** Surgeon to St. Mark’s Ophthalmic Hospital


*Upon the Efficacy and Mode of Administration of Belladonna and Atropia.* By W. R. Wilde, M. R. I. A., Surgeon to St. Mark's Ophthalmic Hospital.—One of the greatest improvements in the oculist's materia medica of late years has been the introduction of the alkaloid denominated Atropia, which, we believe, we were the first to introduce into practice in this city, upwards of two years ago. It was procured for us by Messrs. Bewley and Evans, who have solutions of it according to our formulæ, of the following strengths: one grain, two grains, and three grains to one drachm of distilled water, and three drops of spirits of wine, and numbered 1, 2, and 3. The salt is rendered soluble by the addition of a drop of dilute nitric acid, and the spirit is added to make the solution keep.

A single drop of number 1 placed upon the conjunctiva of the lower lid (where it causes neither pain nor irritation,) in a healthy eye, dilated the pupil, in a period of time varying from five to fifteen minutes, to double, or even more than the ordinary medium size, and will retain it so, upon the average, from four to five days; at which period it generally begins to contract, but the pupil does not fully regain its previous size, nor the iris its mobility, till the sixth or seventh day. There will be, no doubt, exceptions to this rule, particularly in cases where there may be such an idiosyncrasy present as would render the eye susceptible to the action of the atropa belladonna used in any form, and perhaps keep the pupil permanently dilated for months. To counteract this effect of the solution number 1, we have employed upon the second and third day after its application those remedies which generally excite the pupil to contract, such as sudden exposure to strong light, the application of electro-magnetism, the use of opium, and the application of the vinous tincture of that medicine upon the conjunctiva; but each and all these means failed to lessen the size of the pupil, in many instances, until the end of the third, or fourth, or fifth days. Solutions number 2 and 3 produce a more decided effect upon the iris, and in a shorter space of time, and retain the pupil dilated for a longer period—even to the eighth or tenth day. When the object is to keep the pupil in a state of permanent dilatation, as in cases of iritis and aquo-capsulitis, as well as to try and break up recent adhesions between the iris and lens, or to withdraw the iris from protruding through an aperture caused by a wound or ulcer near the centre of the cornea; in all cases of central cataract; where the cornea is opaque in its central portion, or that a portion of the pupillary margin of the iris is attached to the back of the cornea, after the distission of a cataract; or, in fact, in any case in which we wish to produce permanent or complete dilatation of the iris, the solution of atropia will be found much more efficacious than the usual mode of applying the extract of belladonna externally. It is, moreover, much more cleanly, and is not liable to the objection urged against the latter, of producing an unpleasant eruption around the brow on which it is applied, and it is preferable to the ordinary mode of placing a few drops of the solution of the extract between the palpebræ, inasmuch as it causes no pain nor irritation.

In cases, however, where there is much conjunctivitis, or even deeper seated inflammation, attended with lachrymation, present, it does not possess the immediate and marked power over the iris which it does in the healthy eye, and its effects pass off much sooner; and this remark is applicable even to the three-grain solution. It is possible that in such cases the mucous discharge, and particularly the lachrymation which is present, may dilute it too much, or the morbid irritability and increased vascularity of the organ may render it less susceptible of the local application of this remedy than it would be in an otherwise healthy condition, therefore, in cases of violent irritic inflammation, the syphilitic for instance, where the disease had considerably advanced, and extensive exudations of lymph had taken place, we would not solely depend upon the atropia solution, but likewise apply the extract round the orbit. In cases of recent protrusion of the iris through the central portion of the cornea, the result either of injury, or rupture from ulceration, &c., and when there was no great irritability and blepharospasmus present, we have applied the strong atropia solution externally, by means of a small portion of linen rag wet with it, and retained for a short time upon the closed eyelids, with the most happy results.

We may here remark that the benefits arising from dilatation of the pupil have not been sufficiently attended to in the general treatment of ulcers of the cornea. We have, on several occasions lately, been able not only to save the eye, but even to prevent adhesions between the cornea and iris (*synechia anterior*,) and consequent blemish, by means of the judicious application of the preparations of belladonna. In cases of rupture from ulceration, when we have seen the patient shortly after the rupture occurred—and in many of those instances hernia of the iris had absolutely taken place—we at once applied the atropia solution, closed the lids, kept them in that condition with isinglass plaster, and then applied a large pledget of lint smeared with the extract of belladonna over the eye and brow, and retained it in position by a light bandage, at the same time that we employed, when necessary, local depletion by means of leeches on the temples and over the malar bone, together with blistering, and constitutional treatment calculated to lower the inflammation and prevent the further spreading of the sloughy or ulcerative process in the cornea. We keep the eye covered up in this state for thirty-six or forty-eight hours, and have had, in most instances, the satisfaction of finding, when we came to examine the eye, that the iris had been withdrawn from the wound, the pupil had dilated, and the cornea had united.

There are, however, certain cases in which the use of atropia is inadmissible, namely, in examining the eye for cataract, where we do not wish the dilatation of the pupil to continue longer than a few hours, if possible. In cases where we wish to dilate the pupil before we perform the operation for absorption of the lens, we have more than once seen unpleasant consequences result after this manner. It is well known to operative ophthalmic surgeons, that after the dilatation with the ordinary belladonna extract or infusion, the iris will, during the operation of keratonyxis, partially contract, either from the loss of a few drops of aqueous humour, or from its irritability being excited by the side or flat of the needle touching the margin of the pupil, or from the cataractous lens, whole or in a broken condition, pressing against it: and this condition is rather serviceable than otherwise, for should the lens be inclined to start from its bed, and press forward through the pupil into the anterior chamber, the iris acts as a partition to keep it in its place; while in a few hours the aqueous fluid is regenerated, the iris falls back into its natural position, and can afterwards be kept dilated by the continued external application of the belladonna.

If, however, the pupil has been previously dilated by the atropia, it is thoroughly immoveable, and the lens is liable either to press into it or become dislocated, and get into the anterior chamber. This latter accident occurred to us some time since, in breaking up the lens for congenital cataract. We had ordered a solution (No. 2) of atropia to be dropped into the eye the night previously, and on arriving in the morning we found the iris reduced to a mere ring. The child struggled a good deal, and a few drops of the aqueous liquid were lost during the operation, which consisted in a mere crucial incision into the capsule. On withdrawing the needle we remarked that there was no contraction of the pupil, into which the lens pressed. On visiting the child in the evening it had been so uneasy and complained so much of pain, and there was so much lachrymation present, that we were induced to remove the bandage and examine the eye. The cornea was found to have become plump, from the regeneration of the aqueous fluid, but the iris had remained immoveable, and the lens had started into the anterior chamber, where it caused considerable irritation and subsequent inflammation. It absorbed completely, however, without a second operation, and in a much shorter time than usual.

In cases of photophobia following cataract and other operations on the eye, and attended with myosis, which had resisted the continued external application of belladonna, as well as the strong atropia solution dropped into the eye, we have found the most marked beneficial effects result from the internal administration of the extract of belladonna, given in the form of solution, to the amount of the sixteenth of a grain, from three to five times a-day. This, in the course of thirty-six or forty-eight hours has seldom failed to relieve the pain and intolerance of light, and also to dilate the pupil as far as possible.

In neuralgic affections of the eye, characterized by pain of a burning description coming on at a particular, and often regular intervals, sometimes at particular hours of the day, yet induced by reading or using the eye in any fine work, and unattended with inflammation or any apparent alteration in the texture or motion of the organ, &c. &c., in which rest, change of air, tonics of various descriptions, particularly iron, and other means, had failed, we have latterly administered belladonna internally, with the very best effect, in doses varying from the sixteenth to the sixth of a grain three times a-day, given in the form of a solution. It may appear strange, but it is nevertheless true, that in some cases of old and inveterate photophobia, as in that form accompanying pannus, or the ophthalmia attended by vascular cornea in discharged soldiers, the internal use of belladonna will be found most efficacious.

We quote the following from a recent Number of the *Gazette des Hopitaux*: "For a long time M. Berrard has employed in his practice at La Pitie, in place of the extract of belladonna, collyria containing the active principle of belladonna, atropia. This substance, signalized for the first time by M. Brandes, who had not, however, obtained it in its pure state, but since isolated by MM. Meire and Seines, presents many advantages over the extract of belladonna; first, by acting with extreme rapidity in dilating the pupil, and by being endowed with great energy, sufficient to produce its effect in a solution of 0·05 or 0·10, in twenty grammes of distilled water, possibly a consideration of little importance in an hospital, but of great value in private practice in enabling one to avoid the employment of black unctions, which disfigure so much, and for which some patients, particularly females, have a great repugnance."—*Dub. Quart. Journ. of Med. Science.*

